# A Clinical phase I/II trial to investigate preoperative dose-escalated intensity-modulated radiation therapy (IMRT) and intraoperative radiation therapy (IORT) in patients with retroperitoneal soft tissue sarcoma

**DOI:** 10.1186/1471-2407-12-287

**Published:** 2012-07-12

**Authors:** Falk Roeder, Daniela Schulz-Ertner, Anna V Nikoghosyan, Peter E Huber, Lutz Edler, Gregor Habl, Robert Krempien, Susanne Oertel, Ladan Saleh-Ebrahimi, Frank W Hensley, Markus W Buechler, Juergen Debus, Moritz Koch, Juergen Weitz, Marc Bischof

**Affiliations:** 1 Clinical Cooperation Unit Radiation Oncology, German Cancer Research Center (DKFZ), Heidelberg, Germany; 2 Department of Radiation Oncology, University of Heidelberg, Heidelberg, Germany; 3 Department of Surgery, University of Heidelberg, Heidelberg, Germany; 4 Department of Biostatistics, German Cancer Research Center (DKFZ), Heidelberg, Germany; 5 Department of Radiation Oncology, Helios Clinic Berlin, Berlin, Germany; 6 Department of Radiation Oncology, Markus Clinic, Frankfurt, Germany

## Abstract

**Background:**

Local control rates in patients with retroperitoneal soft tissue sarcoma (RSTS) remain disappointing even after gross total resection, mainly because wide margins are not achievable in the majority of patients. In contrast to extremity sarcoma, postoperative radiation therapy (RT) has shown limited efficacy due to its limitations in achievable dose and coverage. Although Intraoperative Radiation Therapy (IORT) has been introduced in some centers to overcome the dose limitations and resulted in increased outcome, local failure rates are still high even if considerable treatment related toxicity is accepted. As postoperative administration of RT has some general disadvantages, neoadjuvant approaches could offer benefits in terms of dose escalation, target coverage and reduction of toxicity, especially if highly conformal techniques like intensity-modulated radiation therapy (IMRT) are considered.

**Methods/design:**

The trial is a prospective, one armed, single center phase I/II study investigating a combination of neoadjuvant dose-escalated IMRT (50–56 Gy) followed by surgery and IORT (10–12 Gy) in patients with at least marginally resectable RSTS. The primary objective is the local control rate after five years. Secondary endpoints are progression-free and overall survival, acute and late toxicity, surgical resectability and patterns of failure. The aim of accrual is 37 patients in the per-protocol population.

**Discussion:**

The present study evaluates combined neoadjuvant dose-escalated IMRT followed by surgery and IORT concerning its value for improved local control without markedly increased toxicity.

**Trial registration:**

NCT01566123

## Background

The retroperitoneal space is the site of origin for about 15% of soft tissue sarcomas [1]. Complete excision with wide margins represents the mainstay of treatment, however even gross total resections are possible only in about 50% of the patients [2,3] because of the often locally advanced tumors which frequently already involve vital structures at time of diagnosis [3]. Even if gross total resection is possible, margins are typically narrow because of the normal tissue limitations [3,4] consequently the local failure rate after surgery alone remains high [5-7]. In accordance to extremity soft tissue sarcomas (STS), where randomized trials have demonstrated improved local control by the addition of radiation [8,9], considerable interest has been paid in the use of postoperative radiation approaches in retroperitoneal STS also. However, the efficacy of postoperative external beam irradiation (EBRT) is limited because of the inability to deliver adequate doses in account for the tolerance limits of stomach, small bowel, kidney, liver and spinal cord [10], especially when conventional radiation techniques are used. Because of the known dose-relationship resulting in improved local control rates if doses beyond 55–60 Gy are used [11,12], some centers including ours investigated the use of an intraoperative radiation therapy (IORT) boost to overcome the dose limits of postoperative EBRT [3,10,13-15]. The only randomized trial reported so far [13] found a significantly improved local control rate of 60% using a combination of 20 Gy IORT and 35–40 Gy EBRT compared to 20% control with 50–55 Gy postoperative EBRT alone. The reduction of local failures was even accompanied by a lower rate of gastrointestinal toxicities with the use of IORT, but neuropathy emerged as a dose-limiting side effect. [13]. However, it has been shown, that neurotoxicity is hardly increased if the intraoperative dose is limited to less than 15 Gy [16]. Given the necessity of a slightly lowered intraoperative dose and the aim of a further improvement in local control, it seems reasonable to investigate an increased EBRT dose component in the combined treatment approach. Compared to the postoperative approach, preoperative radiation therapy and the use of improved irradiation techniques seem favourable for the following reasons : Preoperative radiation therapy allows for a more precise target volume definition and delineation with smaller safety margins, reduces toxicity to adjacent organs at risk because of their displacement through the tumor itself, may lead to a devitalisation of tumor cells including a down-sizing effect, and may avoid a treatment delay due to postoperative complications. Considering improved irradiation techniques, intensity-modulated radiation therapy (IMRT) has been shown to result in improved target coverage and reduced dose to adjacent organs at risk compared to conventional irradiation, as shown in several diseases including retroperitoneal sarcoma [17,18], especially if complex shaped target volumes have to be treated [19]. Further on, IMRT offers the opportunity to reduce overall treatment time using an integrated boost concept with simultaneously increased dose per fraction in parts of the target volume which are at increased risk for incomplete resection during planned surgery.

Therefore, the primary aim of this trial is to investigate the value of dose-escalated preoperative IMRT followed by surgery with an intraoperative electron boost to reduce the local recurrence rate without a markedly increased toxicity. This combination yields total doses which should be able to control even microscopic residual disease without harm to the adjacent organs at risk.

## Methods and design

### Study design

The trial is designed as a prospective single-center one-armed phase I/II study to assess the efficacy and safety of a combination regimen consisting of neoadjuvant intensity-modulated dose-escalated radiation therapy followed by surgery and an intraoperative electron radiation therapy boost to the tumor bed in patients with gross resectable or borderline resectable soft tissue sarcomas of the retroperitoneal space.

### Patient selection and inclusion/exclusion criteria

A minimum of 37 patients fulfilling the inclusion criteria listed below should be enrolled in this trial. Only patients meeting all of the inclusion criteria and missing all of the exclusion criteria (see Table 1) are considered for admission to the trial. Accrual will be stopped after the calculated sample size of the per-protocol population is reached (see statistical considerations).

**Table 1:** Inclusion and exclusion criteria

**Inclusion criteria**	**Exclusion criteria**
written informed consent	missing written informed consent
histologically confirmed, primary or locally recurrent soft tissue sarcoma of the retroperitoneal space	missing histological conformation of soft tissue sarcoma
judged as at least marginally resectable	Desmoid Tumors (aggressive fibromatosis), Gastrointestinal Stroma Tumors (GIST)
absence of distant metastases	judged as gross incomplete or not resectable
tumor size ≥ 5 cm	incomplete staging
	presence of distant metastases
	prior radiation therapy to the abdominal region
	participation in another clinical interventional study
	inflammatory bowel disease

### Trial organization

The study has been designed by the Departments of Radiation Oncology and General Surgery of the University of Heidelberg in cooperation with the Departments of Radiation Oncology, Biostatistics and Medical Physics at the German Cancer Research Center (DKFZ) Heidelberg. It is carried out by the Department of Radiation Oncology at the German Cancer Research Center together with the Departments of Radiation Oncology and General Surgery at the University of Heidelberg. The trial is an investigator initiated trial.

### Coordination

The trial is coordinated by the Department of Radiation Oncology at the German Cancer Research Center (DKFZ) in cooperation with the Departments of General Surgery and Radiation Oncology of the University of Heidelberg. The Departments of Radiation Oncology at the German Cancer Research Center and at the University of Heidelberg are responsible for overall trial management, database management, quality assurance, reporting and for the scientific program of all trial related meetings. The trial has been registered at ClinicalTrials.gov [NCT01566123].

### Investigators

Patients will be recruited by the Departments of General Surgery, Radiation Oncology or Internal Medicine at the University Hospital of Heidelberg, the Department of Radiation Oncology at the German Cancer Research Center or the National Center for Tumor Diseases (NCT) at the University of Heidelberg. All investigators are experienced oncologists from the field of radiation oncology or surgery cooperating in this trial.

### Ethical and legal considerations

The study protocol was approved by the independent ethics committee of the Medical Faculty at the University of Heidelberg. The trial is carried out by adhering to local legal and regulatory requirements. The study complies with the Declaration of Helsinki 2004, the principles of Good clinical practice (GCP) and the German Federal Data Protection Act. Written informed consent is obtained from each patient before inclusion into the trial after nature, scope and possible consequences of participation in the trial have been explained by a physician.

### Study objectives and endpoints

The primary objective is the local control rate after 5 years. Secondary objectives are progression-free-survival, overall survival, acute and late toxicity, resectability and patterns of recurrence.

### Pretreatment evaluation

Initial work up consists of clinical examination, laboratory tests including renal and hepatic function, histological confirmation of diagnosis, CT or MR-imaging of the abdominal cavity, thoracic CT, bone scan, scintirenography, evaluation of general ability to receive major surgery and evaluation of resectability.

### Treatment assignment and schedule

**Figure 1:**
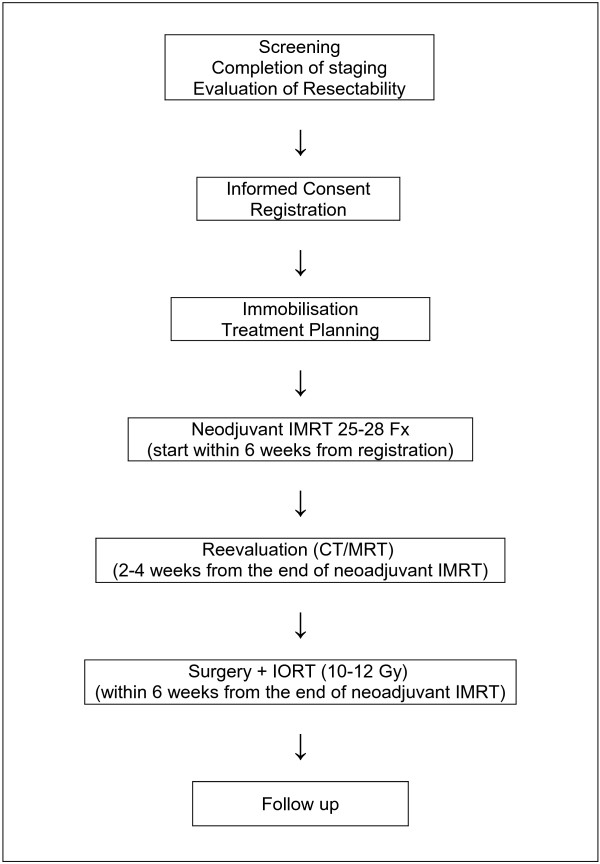
**Flow Chart of the Study.** IMRT: Intensity-Modulated Radiation Therapy, IORT: Intraoperative Radiation Therapy, Fx: Fractions.

All patients eligible for the trial after pre-treatment evaluation (see inclusion/exclusion criteria, Table 1) who gave written informed consent are registered in the trial and assigned to the same treatment regimen (see Figure 1). They receive immobilization and treatment planning examinations for neoadjuvant intensity-modulated radiation therapy (IMRT) either at the Department of Radiation Oncology at the German Cancer Research Center or the University of Heidelberg. Neoadjuvant intensity-modulated irradiation starts within 6 weeks after registration and is carried out in 5 fractions per week for 5–6 weeks using an image guided, stereotactic, intensity-modulated approach up to a total dose of 50–56 Gy. Reevaluation including restaging and assessment of toxicity takes place within 2 to 4 weeks after the last fraction. Surgical resection of the tumor including Intraoperative Radiation Therapy (IORT) with a single dose of 10–12 Gy is carried out within 6 weeks after the end of neoadjuvant irradiation at the Department Surgery in cooperation with the Department of Radiation Oncology at the University of Heidelberg. After postoperative recovery, all patients will be assigned to regular follow up visits either at the Department of Radiation Oncology at the German Cancer Research Center or at the Departments of Surgery or Radiation Oncology.

### Neoadjuvant intensity-modulated radiation therapy and reevaluation

For external beam radiation therapy, patients are immobilized using a custom-made, individually manufactured body mask system (Scotch Cast^TM^, 3 M, St. Paul-Minneapolis, USA) or a vacuum pillow (BlueBag^TM^, Medical Intelligence, Schwabmünchen, Germany) combined with a custom-made individually manufactured head mask system (Scotch Cast^TM^, 3 M, St. Paul-Minneapolis, USA) mounted to a stereotactic body frame, resulting in an accuracy of less than 3.6 mm [20]. For treatment planning, contrast enhanced CT as well as MR imaging is performed for optimal target definition. Organs at risk like small bowel, stomach, liver, kidneys, bladder, femoral heads and spinal cord are contoured. The Gross Tumor Volume (GTV) includes all macroscopically visible tumor lesions on imaging. The clinical target volume (CTV) includes the gross tumor volume with a safety margin of 1.5 cm in all directions. A safety margin of 5 mm is added to obtain the Planning Target Volume (PTV). The margins may be reduced with respect to anatomical borders or organs at risk at the discretion of the investigator. The prescribed dose to the PTV is 45–50 Gy in single fractions of 1.8-2.0 Gy. The prescribed dose to the GTV is 50–56 Gy in single fractions of 2.0-2.4 Gy using an integrated boost concept. The total dose is prescribed to the median of the GTV, while the target volumes should be surrounded by the corresponding 95% isodose lines. The generally accepted tolerance doses [21] for the organs at risk are maintained during the radiation therapy planning. Inverse treatment planning is carried out using the planning systems at the Department of Radiation Oncology of the German Cancer Research Center. Treatment is performed using step-and-shoot intensity modulated radiation therapy with 5 to 13 coplanar 6 MV-beams after stereotactic target point localisation. Setup correction is done using an image-guided approach with an In-Room-CT on rails (Siemens Somatom Emotion, Siemens, Erlangen, Germany) by comparison of the current CT with the planning CT at least once a week. Reevaluation including restaging with abdominal CT or MRI and assessment of toxicity is performed 2 to 4 weeks after the last fraction of neoadjuvant irradiation.

### Surgery and intraoperative radiation therapy

Surgery is performed within six weeks after completion of neoadjuvant irradiation at the Department of General Surgery of the University of Heidelberg. A gross total resection is attempted. An additional intraoperative electron boost will be performed using a dedicated linear accelerator inside the operation theatre (Siemens Mevatron, Siemens, Concord, USA). The target volume of the intraoperative boost includes the tumor bed or the high risk region for positive or close margins as defined by the treating surgeon and the radiation oncologist together if the complete tumor bed cannot be covered. Therefore an applicator of appropriate size is placed inside the abdominal cavity and attached to the table. Uninvolved radiosensitive tissues are removed from the treatment area or covered by lead shielding. After alignment with the accelerator, the target volume is irradiated with a single dose of 10–12 Gy, prescribed to the 90% isodose. The electron energy is selected according to the tissue depth that has to be covered in order to encompass the target volume with the 90% isodose.

### Follow up

Regular follow up visits start at the day of discharge from the Department of General Surgery at the University of Heidelberg and will take place every 3 months after surgery for the first 2 years and every 6 months for three further years. They will include clinical and laboratory examinations, CT or MR-imaging of the abdominal cavity, thoracic CT (every 6 months in the first two years, every year thereafter) and assessment of acute and late toxicity as well as any other necessary examination, test or imaging in case of suspicion of local or distant failure at discretion of the treating physician. All patients will be followed for 5 years or until death or end of study participation due to other reasons.

### Analysis populations

#### Full analysis set

The Full analysis Set (FAS) consists of all patients included in the trial irrespective whether any protocol violation was present during treatment under study conditions or whether the patient was taken off-study any time after treatment start, except patients who withdraw informed consent before the first radiation treatment or about whom it becomes known that major in-/exclusion criteria have been violated which would have excluded them from study participation when known at start of treatment.

#### Per protocol population

The Per Protocol population consists of all patients meeting the inclusion criteria and receiving the full treatment according to this protocol.

### Assessment of efficiacy

Local control rate after 5 years is the primary endpoint of the trial. It will be assessed by repeated CT or MR-imaging during regular follow up. In case of a single suspicious locoregional lesion, histological confirmation will be attempted. Otherwise, new lesions with typical radiological signs and/or clinical behaviour of a local recurrence will be counted as local recurrence. In case of missing surgical resection of the tumor after neoadjuvant radiotherapy, a local disease progression according to RECIST criteria (version 1.0) will be counted as a local recurrence. Special attention should further be given to avoid that tissue reaction to radiation or surgical treatment is classified as local recurrence or disease progression. Variations in post-radiotherapy or post-surgery imaging may continue for months and may be accompanied by clinical signs and symptoms. In such cases, the labelling of the lesion suspicious for recurrence or progression must be based on the clinical follow-up including their developement over time. If the course of events shows that true recurrence or progression indeed occurred, the date of the first appearance or progression of the suspicious lesion is to be considered as the date of recurrence or progression.

Progression-free survival (PFS) is a secondary endpoint of the study, determined as the time span from the first day of radiation therapy until local or distant recurrence or progression or death due to any cause is found, whatever occurred first. Patients alive without progressive disease at the time of data analysis will be censored at the time of the most recent follow up. In case of lesions suspicious for distant metastases, histological or cytological confirmation is attempted. Otherwise, new lesions with typical radiological signs and/or clinical behaviour of distant metastases will be counted as progression.

Overall survival is a secondary endpoint of the study, calculated from the first day of radiation treatment until death of any cause. Patients not reported dead or lost to follow-up will be censored at the date of the last follow-up examination.

### Assessment of toxicity

Acute radiation toxicity will be assessed according to Common Terminology Criteria for Adverse Events Version 3.0 (CTCAE 3.0) during the time period from the first day of neoadjuvant radiation treatment until 3 months after surgery. Late radiation toxicity will be scored according to CTCAE 3.0 and RTOG criteria. Toxicity will regularly be evaluated by clinical and laboratory examinations at least once a week during neoadjuvant external beam radiation therapy and the first follow up visits at discharge of the Department of General Surgery and after 3 months. Late toxicity will regularly be scored during every further regular follow up visit.

### Sample size estimation, confirmatory and descriptive analyses

The primary endpoint of the trial is the local control rate after five years (LC-5yR). The study is designed to demonstrate that the combination of neoadjuvant dose-escalated intensity-modulated radiation therapy with surgery and intraoperative radiation therapy can improve the local control rate after five years. Local control rate after five years in a comparable patient population treated with standard procedures (surgery followed by adjuvant three-dimensional conformal radiotherapy) was estimated to be 50% according to the literature. The sample size calculation was designed on the assumption to detect an improvement by 20% in this primary endpoint. For sample size calculation, the following hypotheses have been made:

p0 is the largest LC-5yR which, if true, implies that the efficacy of the study treatment is too low. In the present trial P0 has been taken as 50%

p1 is the lowest LC-5yR which, if true, implies that the efficacy of the treatment is adequate. In the present trial p1 has been taken as 70%

α is the acceptance probability of considering adequate efficacy of the treatment with a true LC-5yR equal or lower to p0 (false positive decision). In the present trial α has been taken as 5%

β is the acceptance probability of rejecting adequate efficacy of the study treatment with a true LC-5yR at least equal to p1 (false negative decision). In the present trial β has been taken as 20%.

Using the one-sided binomial test (“Exact Test”) with the given hypotheses, the study requires 37 patients to decide whether the LC-5yR is less or equal to 0.5 or greater or equal to 0.7. If the number of locally controlled patients is ≥24, the hypothesis that LC-5yR is ≤ 0.5 will be rejected with a target error rate of 0.05. Sample size calculation and confirmatory analysis are based on the per protocol population [22].

Secondary endpoints are all of explorative nature and reported using descriptive analysis methods. Time to event data (progression free survival, overall survival) will be evaluated using the Kaplan-Meier-Method.

### Safety and discontinuation of treatment

Toxicities are classified by type, grade, duration, onset and relationship to radiation treatment. Severe acute gastrointestinal toxicity (≥ grade 3) is the main dose limiting factor in patients receiving adjuvant three-dimensional conformal radiation therapy after surgery. The safety analysis was designed to demonstrate that the combination of neoadjuvant intensity-modulated radiation therapy with surgery and intraoperative radiation therapy will not result in an increased rate of severe acute gastrointestinal toxicity. The rate of patients who will suffer from acute severe gastrointestinal toxicity in a comparable patient population treated with standard procedures (surgery followed by adjuvant three-dimensional conformal radiotherapy) was estimated to be 20%.

A toxicity related discontinuation criteria was implemented such that after having recruited about one third of the total number of patients an increase by 20% of the onset of severe acute gastrointestinal toxicities (from 20% to 40%) should be detected with a power of 80% at a level of significance of α = 5% in one interim analysis. Therefore, an optimal two-stage design [23] was calculated which suggested to do an interim analysis after n1 = 15 patients among a total of 43 and to stop for toxicity when more than r1 = 5 patients would show severe gastrointestinal toxicity. Since the calculated sample size was n = 37, these numbers were adjusted to n1 = 13 and r1 = 4. Therefore, the study should stop for toxicity after 13 patients if ≥ 5 patients would have developed a severe acute gastrointestinal toxicity.

### Current status

To date 20 patients have been enrolled so far. The planned safety analysis has taken place after the first 13 patients enrolled on the study had completed local treatment:

Severe acute gastrointestinal toxicity ≥ grade III was observed in 1 patient (lymph fistula and duodenal stenosis after surgery with the need for parenteral nutrition until stent placement). Other acute severe toxicities ≥ grade III during radiation therapy were found in 1 patient (leucopenia). Other severe postoperative complications defined by the need for reintervention or intensive care treatment occurred in 2 of 13 patients (acute renal failure and sepsis in one and severe wound healing disturbance in one patient). No fatal toxicities (grade 5) have been observed so far. As the toxicity related discontinuation criteria (see safety and discontinuation of treatment) was not reached, the investigators decided to continue with the study until the planned sample size will be reached.

## Competing interests

The authors declare that they have no competing interests.

## Authors’ contributions

FR prepared the manuscript and participated in conducting the study. PEH assisted in preparing the manuscript, conducting the study and reviewed the manuscript. LE performed the biostatistical parts of the study design, prepared the corresponding parts of the study protocol and assisted in preparing the manuscript. FWH assisted in study conception, preparation of the study protocol and the manuscript and participated in conducting the trial regarding the aspects of radiation physics. DSE planned the study, prepared the study protocol, conducted the correspondence with the ethics committee and participated in study conduction. AN assisted in planning of the study, preparation of the study protocol and participated in conducting the study. SO, RK and MK participated in conducting the study. LSE participated in the safety analysis and assisted in preparing the manuscript. MWB and JD participated in study conception and design. JW planned the study regarding surgical aspects, prepared the corresponding parts of the study protocol, and conducted the surgical part of the study. MB participated in manuscript preparation, conduction of the study and reviewed the manuscript. All authors read and approved the final manuscript.

## Pre-publication history

The pre-publication history for this paper can be accessed here:

http://www.biomedcentral.com/1471-2407/12/287/prepub
